# Genetic copy number variants in sib pairs both affected with schizophrenia

**DOI:** 10.1186/1423-0127-17-2

**Published:** 2010-01-11

**Authors:** Chia-Huei Lee, Chih-Min Liu, Chun-Chiang Wen, Shun-Min Chang, Hai-Gwo Hwu

**Affiliations:** 1 National Institute of Cancer Research, National Health Research Institutes, Zhunan Town, Miaoli County 350, Taiwan; 2 Department of Psychiatry, National Taiwan University Hospital and National Taiwan University College of Medicine, Taipei, Taiwan; 3 Institute of Epidemiology, College of Public Health, National Taiwan University, Taipei, Taiwan; 4 Department of Psychology, College of Science, National Taiwan University, Taipei, Taiwan

## Abstract

**Background:**

Schizophrenia is a complex disorder with involvement of multiple genes.

**Methods:**

In this study, genome-wide screening for DNA copy-number variations (CNVs) was conducted for ten pairs, a total of 20 cases, of affected siblings using oligonucleotide array-based CGH.

**Results:**

We found negative symptoms were significantly more severe (p < 0.05) in the subgroup that harbored more genetic imbalance (n ≧ 13, n = number of CNV-disrupted genes) as compared with the subgroup with fewer CNVs (n ≦ 6), indicating that the degree of genetic imbalance may influence the severity of the negative symptoms of schizophrenia. Four central nervous system (CNS) related genes including CCAAT/enhancer binding protein, delta (*CEBPD*, 8q11.21), retinoid × receptor, alpha (*RXRA*, 9q34.2), LIM homeobox protein 5 (*LHX5*, 12q24.13) and serine/threonine kinase 11 (*STK11*, 19p13.3) are recurrently (incidence ≧ 16.7%) disrupted by CNVs. Two genes, *PVR *(poliovirus receptor) and *BU678720*, are concordantly deleted in one and two, respectively, pairs of co-affected siblings. However, we did not find a significant association of this *BU678720 *deletion and schizophrenia in a large case-control sample.

**Conclusions:**

We conclude that the high genetic loading of CNVs may be the underlying cause of negative symptoms of schizophrenia, and the CNS-related genes revealed by this study warrant further investigation.

## Background

Schizophrenia is a devastating mental disorder, and its etiology has yet to be fully elucidated. Genetic epidemiological studies have shown that schizophrenia is predominantly genetically determined and has a high heritability, with a multi-locus inheritance model [1]. Chromosomal abnormalities occurring in patients with schizophrenia may provide useful information for locating and fine mapping the relevant gene loci. This has been demonstrated by the identification of the potential vulnerability genes of proline dehydrogenase (PRODH) [2] and Disrupted-in-Schizophrenia 1 (DISC1) [3,4] based on 22q11 micro-deletion syndrome and balance translocation (1;11) (q42.1;q14.3), respectively.

Although linkage studies in schizophrenia have provided some evidence of susceptible loci over many broad chromosomal regions, pinpointing causative gene mutations by conventional linkage strategy alone is problematic [5]. On the other hand, the resolution, typically ranging from 5 to 10 Mb [6,7], of traditional cytogenetic techniques such as fluorescence in situ hybridization (FISH), comparative genomic hybridization (CGH), and spectral karyotyping (SKY)-FISH is limited, and submicroscopic aberrations (fewer than a few tens of kilobases) of genomic DNA may be impossible to identify using these methods. Due to the technical constraints, we believe that the importance of chromosomal anomalies that could be the major cause of schizophrenia may have been overlooked in the past.

One of the chromosomal alterations involving amplifications and deletions of genetic materials is referred to as a copy number variant (CNV). Because of the rapid development of molecular genetic tools, recent studies have demonstrated the presence of several CNVs within the human genome. Some genomic CNVs called copy number polymorphisms (CNPs) may not be pathogenic and simply contribute to human genetic diversity and individual variability in response to environmental stimuli [8-10]. On the other hand, some CNVs have proven to be associated with several human diseases, including cancer [11], intellectual disability [12-14], and autism [15]. These discoveries have encouraged investigators to study CNVs in complex disorders like schizophrenia. Using the array-based comparative genomic hybridization (array CGH) technology, many genome-wide surveys of CNVs implicated in schizophrenia have been completed [16-24]. Three large-scale studies [17,19,22] suggested that rare copy number alterations collectively are significant risk factors for this disease. These works also revealed that specific CNVs on chromosomes 1 and 15 were responsible for vulnerability to schizophrenia. In addition, Kirov's work [24] suggested that a deletion at 2p16.3 disrupting NRXN1 and a duplication at 15q13.1 spanning APBA2 may be implicated in schizophrenia. Even so, a large fraction of the overall genetic risk for schizophrenia remains unexplained.

The present study explored the CNVs in genomic DNA of familial schizophrenia, assuming that the higher genetic loading in schizophrenic families may reveal significant copy number aberrations. Thus, we studied co-affected siblings to highlight the influence of predisposing genetic components and expected to find concordant CNVs in them.

## Methods

### Recruitment of healthy controls

Healthy controls were recruited from the employees of the National Taiwan University Hospital. After signing informed consent, the individuals underwent a screening interview followed by blood withdrawal. Exclusion criteria for the healthy controls were: under age 30; diagnosed with psychiatric disorder, especially schizophrenia; having a history of diabetes mellitus (DM), major systemic disorder, or neurological disorder (e.g., epilepsy); mental retardation; facial dysmorphism; and clinical evidence of brain, trunk or limb anomalies.

### Establishment of control genomic DNA pool

The control DNA pools were constructed by pooling equal amounts of DNA extracted from ten healthy men and ten healthy women. These normal genomic DNA pools were used as reference samples for array CGH analysis and real-time quantitative PCR.

### Recruitment of schizophrenics

Patients for array CGH analysis were enrolled from the outpatient clinics of the Department of Psychiatry, National Taiwan University Hospital. The inclusion criteria were: a diagnosis of schizophrenia according to the Diagnostic and Statistical Manual of Mental Disorders, 4^th ^edition (DSM-IV) [25] and confirmed by the Diagnostic Interview for Genetic Study (DIGS) and at least two siblings affected by schizophrenia in a given family. Patients affected with mental retardation, facial dysmorphysm, or clinical evidence of brain, trunk or limb anomalies were excluded. A total of ten pairs of schizophrenic siblings, a total of 20 cases, from ten unrelated Taiwanese families (A-J), were recruited. The mean age of the subjects was 30.6 years.

After the homozygous deletion of *BU678720 *was found in the initial 20 subjects, a different sample was recruited between 2003 and 2005 in Taiwan for genetic study. A total of 107 controls, 163 simplex schizophrenic patients (those from families with only one member affected with schizophrenia), and 72 multiplex schizophrenic patients (those from families having at least two affected siblings) were included in this study. All cases fulfilled the DSM-IV criteria for schizophrenia.

### Assessment of clinical psychopathology for schizophrenia

Clinical symptoms were rated using the scale for the assessment of negative symptoms (SANS) [26] and the scale for the assessment of positive symptoms (SAPS) [27], both of which have demonstrated satisfactory reliability. According to SANS, the negative symptom score was the sum of scores for Affective Blunting, Alogia, Avolition-Apathy, and Anhedonia-Asociality. The Continuous Performance Test (CPT) [28] and Wisconsin Card Sorting Test (WCST) [29] were used for neuropsychological assessment of sustained attention and executive function, respectively. The assessment methods were described in our previous reports [28,29].

### Genomic DNA extraction

Genomic DNA was isolated from peripheral blood lymphocytes with the PureGene DNA Purification Kit (Gentra Systems, Minneapolis, MN, USA) according to the manufacturer's instructions.

### Array-based CGH (array CGH) experiment

A commercial oligonucleotide array (Human Genome CGH microarray 44B, Agilent Technologies, Palo Alto, CA, USA) was used for array-CGH analysis. Genomic DNA fragmentation, labelling and array hybridization were performed as previously described [30,31]. Each array hybridization experiment was performed with differentially labelled gender-matched samples, one from the affected individual, and the other from the DNA control pool. To rule out probable CNPs in our ethnic group, two array hybridization experiments were performed using the male or female pooled control and commercial, normal, same-gender Caucasian samples (Promega, Madison, WI, USA).

### Selection of high-confidence copy number alterations

Filtering procedures were applied to select qualified data sets for analysis. In total, 18 qualified arrays, exclusive of the samples for patients F2 and J2, were selected for further analysis. Aberrations were only considered if the aberration scores, automatically generated by Agilent CGH analysis software, were higher than 1.00 or lower than -1.00. The CNVs which exist within the control genome and are unlikely to be pathogenic were filtered out by comparison to the CNVs identified from the two reference arrays which were performed with normal male and female DNA pools in our laboratory. Additionally, the published CNVs listed in the Database of Genomic Variants [32] with relative high incidence in control subject were also excluded.

The data discussed in this publication have been deposited in NCBI's Gene Expression Omnibus (GEO) [33] and are accessible through GEO series accession number GSE16930.

### Examination of array CGH findings

In order to examine the copy number changes of those genes found in our array CGH analysis, we used the quantitative real-time PCR method. The detailed qPCR conditions were described by Lee [31]. The specific oligonucleotide primer pairs were selected from the Universal Probe Library (Roche Molecular Systems, Inc., Branchburg, NJ, USA). The ATPase, Ca++ transporting, plasma membrane 4 (*ATP2B4*, NM_001001396) was chosen as the reference. The fold change in gene copy number for a target gene is calculated by using the comparative ΔC_T _method as described previously [34]. In each experiment the samples were analyzed in triplicate. The primer information is provided as Supplementary Table 1 (Additional file 1).

**Table 1:** Classification of schizophrenic patients according to the sum of copy-number altered loci^a ^identified by array-CGH.

	Case ID	Number of Deleted Loci	Number of Amplified Loci	Total Number of Altered Loci
Subgroup I	A1	4	2	6
	B2	2	-^b^	2
	C1	1	2	3
	E1	-^b^	-^b^	-^b^
	F1	-^b^	1	1
	G1	3	-^b^	3
	G2	1	-^b^	1
	J1	2	1	3

Subgroup II	A2	10	3	13
	B1	34	-^b^	34
	C2	317	1	318
	D1	86	-^b^	86
	D2	2	28	30
	E2	34	-^b^	34
	H1	90	-^b^	90
	H2	2	14	16
	I1	41	-^b^	41
	I2	25	-^b^	25

^a ^the sum of copy-number altered loci included all aberrant probes assigned to the functional annotated genes, anonymous genes, and intergenic sequences.

^b ^No CNV was detected.

In order to investigate the prevalence of homozygous deletion of the specific *UB678720 *allele revealed in this array CGH study in a different sample, we used PCR followed by electrophoresis. PCR was carried out with the same primers as those used for qPCR. Details of PCR conditions were as described elsewhere [31]. Amplified products were analyzed by on-chip electrophoresis using Agilent 2100 bioanalyzer and Agilent DNA 1000 LabChip kit (Agilent Technologies). Homozygous deletion of *BU678720 *was readily distinguishable by the presence of the 85 bp amplified fragment.

### Data analysis methods

For array CGH analysis, the hybridized arrays were scanned and analyzed as previously described [30,31]. Briefly, after washing, the hybridized arrays were immediately scanned at a resolution of 5 μ using an Agilent G2565BA DNA microarray scanner. The microarray images were analyzed using Agilent Feature Extraction software, version 8.1.1. Another custom analytical software package, Agilent CGH Analytics, version 3.4, was used for the subsequent data analysis. The locations of the copy number aberrations were calculated using the Aberration Detection Method 2 (ADM2) statistical algorithm. The ADM2 threshold was set at 9.0 to make an amplification or deletion determination. According to these settings, the aberration score was generated automatically for each altered locus.

The comparison of the negative symptoms between subgroups was calculated by using the Mann-Whitney U test. The comparison of the incidence of BU678720 homozygous deletion between multiplex and simplex families was analyzed by using the Genmod procedure with software SAS 9.1.

## Results

### Classification of patients with schizophrenia

A total of 379 loci disrupted by CNVs were found. These included 343 losses, 10 gains, and 26 with both losses and gains. Summaries for each patient are presented in Table 1. There were great variations in the number of loci affected by CNVs (range 0-318). Thus we classified patients into subgroups based on the number of loci with CNVs. There were six or fewer scattered loci disrupted by CNVs in subgroup I (A1, B2, C1, E1, F1, G1, G2, and J1), while subgroup II had up to 318 loci with CNVs (A2, B1, C2, D1, D2, E2, H1, H2, I1 and I2). We also noted that the patterns of CNVs showing deletions were more common than those showing amplifications. In addition, we observed that four sib-pair subjects (50%) were in different subgroups, while there were four sib-pair subjects (50%) in the same subgroup (one in subgroup I, three in subgroup II) (Table 1).

### Comparison of psychopathological parameters

We compared the clinical features between subgroup I and subgroup II patients and examined whether the array CGH profiles were correlated with the phenotypes defined by psychopathological parameters of clinical symptoms and neuropsychological performance. We found that the negative symptom score was significantly higher (Mann-Whitney *U *= 14, n1 = 8, n2 = 9, p = 0.033, two-tailed) for subgroup II when compared to subgroup I (Table 1). However, there was no significant difference in the scores for delusions/hallucinations and disorganized symptoms dimensions. There were also no differences in the performance of sustained attention and executive function between these two subgroups.

### Potential candidate genes revealed by array CGH

The genes related to CNS growth and development were referred to as CNS-related from a computer search of the relevant literature on PubMed [35]. For subgroup I patients, the three CNS-related aberrant loci including BTB (POZ) domain containing 8 (BTBD8, NM_183242, 1p22.1), paired-like homeobox 2b (PHOX2B, NM_003924, 4p12) and apoptosis-associated tyrosine kinase (AATK, NM_001080395, 17q25.3) were detected in patients A1, G2 and F1, respectively. It was noted that the copy number gain of AATK and copy number loss of PHOX2B were the only CNVs detected in patients F1 and G2, respectively.

**Figure 1:**
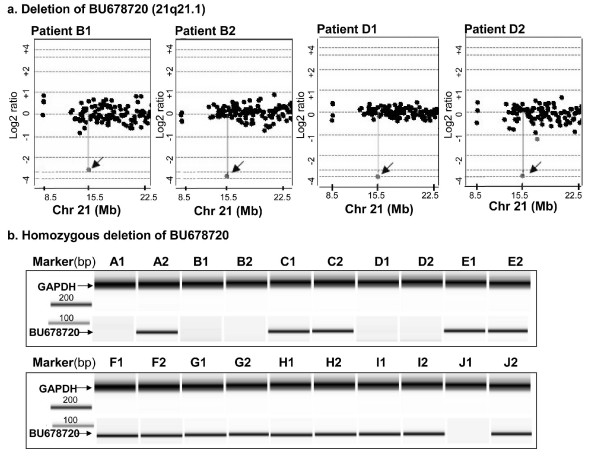
**Concordant loss of BU678720 in sib-pairs of schizophrenics**. (a) Array CGH profiles are shown for patients B1, B2, D1 and D2. The X axis marks the chromosome coordinate in megabases. The Y axis marks the hybridization ratio plotted in log_2 _scale. In these four patients, there is strong indication of a loss of BU678720 (Arrows). Graphics are produced by using Agilent software CGH Analytics version 3.4. (b) LOH analysis of BU678720. Gel view of the PCR products of BU678720 and GAPDH were shown for all affected sib-pairs. The specific amplified fragments of BU678720 and GAPDH were indicated. Graphics are generated by Agilent Bioanalyzer.

Of the genes disrupted by CNVs identified in the subgroup II patients (10 subjects), a total of 16 genes with an incidence of at least 30% (3/10) were selected. The distributions of these highly recurrent CNVs are presented in Table 2. Four of these 16 target loci, genes encoding CCAAT/enhancer binding protein, delta (*CEBPD*, NM_005195), retinoid × receptor, alpha (*RXRA*, NM_002957), LIM homeobox protein 5 (*LHX5*, NM_022363), serine/threonine kinase 11 (*STK11*, NM_000455) are CNS-related. There are only two genes, *PVR *and BU678720, with familial incidence. Of these two genes, the *BU678720 *(BU678720) (function not yet known) had a concordant loss in both sib-pairs of families B and D (Figure 1) and accounted for the highest familial incidence (Additional file 2: Supplementary Table 2). In comparison with healthy controls determined by screening against the Database of Genomic Variants, the alteration incidences are rather high in schizophrenics for all 16 identified CNVs (Additional file 2: Supplementary Table 2, Table 2, Fig 1).

**Table 2:** The highly recurrent CNVs in schizophrenic subjects

Cytoband	Gene symbol	Aberration scores^a^							
		
		A2	B1	C2	D1	D2	E2	H1	I1
7q36.1	PRKAG2			-3.34	-3.25		-3.72		-2.94
8q11.21	**CEBPD**		-2.75		-3.68		-3.59		-3.03
9q31.2	KLF4		-2.58	-2.53	-3.55		-3.42		
9q34.2	**RXRA**			-3.12	-1.71		-1.67	-3.26	-2.53
	UBADC1		-1.73		-3.54				-3.05
	LCN6	-2.21	-1.97		-2.14		-3.27		-2.19
9q34.3	LCN8	-2.21	-1.97		-2.14		-3.27		-2.19
	C9orf37			-1.22		-3.07	3.09		
10q22.1	COL13A1		-1.52	-3.65	-3.3				
12q24.13	**LHX5**	-4.11	-3.79				-3.23		
15q26.1	RHCG	-4.02	-4.26				-3.45		
17q25.2	SEPT9			-3.31	-3.34		-3.24		
19p13.3	**STK11**			-3.07				-2.68	-2.43
19q13.31	PVR			-3.09	-2.44	3.24			
21q21.1	BU678720		-2.79		-4.03	-3.81			
21q22.3	C21orf57			-3.85	-3.22				-2.94

^a ^The aberration scores (n) are calculated by using the Agilent aberration detection method: no change is defined as n = 0, loss as n < -1, and gain as n > 1. Bold type indicates CNS-related genes. Description and GenBank accession no.: Protein kinase, AMP-activated, gamma 2 non-catalytic subunit (PRKAG2 , NM_001040633), Kruppel-like factor 5 (KLF4, NM_001730); UBA domain containing 1 (UBADC1, NM_016172); lipocalin 6 (LCN6, NM_198946); lipocalin 8 (LCN8, NM_178469); chromosome 9 open reading frame 37 (C9orf37, NM_032937); collagen, type XIII, alpha 1 (COL13A1, NM_001130103); Rh family, C glycoprotein (RHCG, NM_016321); septin 9 (SEPT9, NM_001113496); poliovirus receptor (PVR, NM_006505); and chromosome 21 open reading frame 57 (C21orf57, NM_058181).

### Examination of array CGH findings

Quantitative PCR analysis was performed for the four recurrent CNS-related genes, *CEBPD*, *RXRA*, *LHX5*, *STK11*, and the gene of *BU678720 *with a high incidence (two out of 8 pairs: 25%) of concordant loss in both sib-pairs. We analyzed the siblings for whom CNS-related CNVs were detected in at least one of the sib pairs. Comparisons between results from arrays and those from qPCR are summarised in Supplementary Figure 1 (Additional file 3). Deletions of BU678720 could be confirmed by qPCR, while, for others, the qPCR patterns were not perfectly consistent for array CGH results. For BU678720, the low qPCR signals suggested that the gene copy may be completely lost in sib pairs from families B and D.

**Figure 2:**
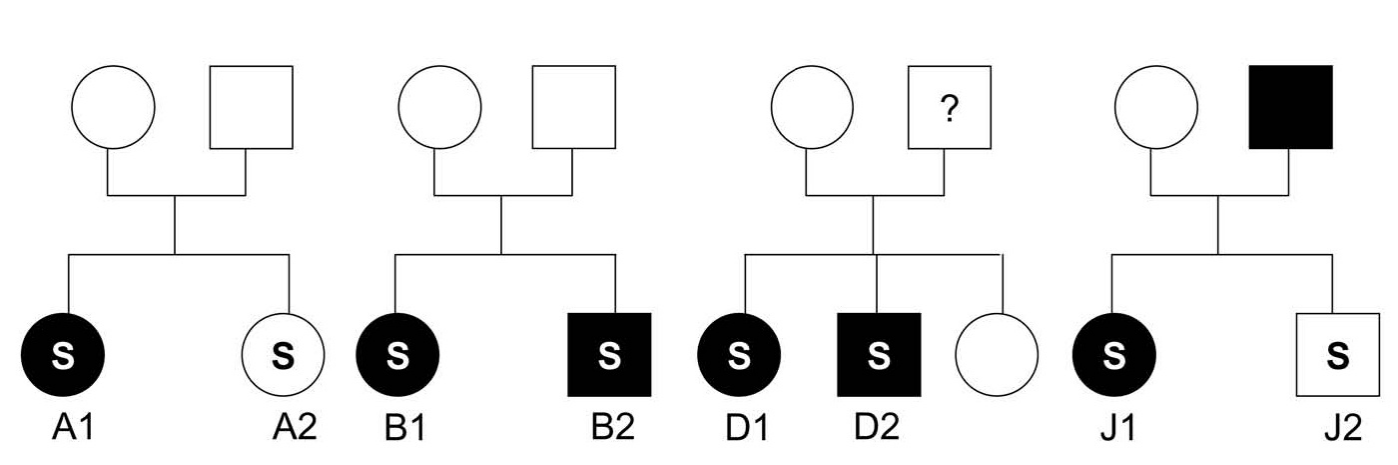
**Pedigree of family and genotyping by BU678720 LOH**. "S" stands for individuals with schizophrenia, the filled symbols indicate individuals with BU678720 LOH and open symbols the individuals without BU678720 LOH. The question mark indicates an individual in whom LOH analysis was not performed.

Since homozygous deletion may have a more profound influence on regulation of gene expression than single copy deletion, we performed the PCR experiments on the ten pairs of affected siblings to detect a possible homozygous deletion in the four CNS-related candidate genes (CEBPD, RXRA, LHX5 and STK11) and the gene BU678720. Homozygous deletion was detected in the gene BU678720 in six individuals A1, B1, B2, D1, D2, and J1 (Fig. 1b), while no homozygous deletion was detected in the four CNS-related candidate genes. To assess whether the homozygous deletion cosegregated with schizophrenia in the four families, we recruited the first-degree relatives of families A, B, D and J to examine for homozygous deletion of BU678720. The results are depicted in Figure 2. Homozygous loss of BU678720 cosegregated with schizophrenia in families B and D. However, the phenomenon of cosegregation was not observed in families A and J. To further clarify the association between BU678720 and schizophrenia, we performed the PCR assay in 72 and 163 affected individuals from multiplex and simplex families, respectively, as well as in 107 controls. The homozygous deletion of BU678720 was detected in 7.4% of patients from simplex families, 11.1% of patients from multiplex families, and 8.4% of controls. Though the prevalence in the patients from the multiplex families was higher than that in controls and patients for simplex families, the comparisons did not attain a level of statistical significance (p = 0.098, df = 1).

## Discussion

Schizophrenia is known as an etiologically diverse psychiatric disorder which exhibits both familial (hereditary) and nonfamilial (sporadic) patterns. To increase the possibility of discovering genomic numerical alterations that contribute to the genetic component of schizophrenia, we recruited 10 affected sibling pairs, a total of 20 affected subjects, for a genome-wide study with the aid of commercial CGH oligonucleotide array. There are conspicuous differences which discriminate our experimental design from those of similar recent investigations: predisposing genetic aberrations as the major causative factor have been emphasized in our study sample of ten affected sibling pairs, instead of sporadic patients without a positive family history of schizophrenia; and, the use of oligoarray (average spatial resolution approximately 35 Kb) instead of BAC array for array CGH has the potential to directly indicate the genes associated with schizophrenia.

The prevalence of concordant CNVs in pairs of co-affected siblings seems low. We found that only two genes, PVR and BU678720, had concordant deletions in both affected siblings with 12.5% (1/8) and 25% (2/8), respectively, of familial incidence. Using three different experiment designs of array CGH, qPCR and PCR, we found that the homozygous deletion of *BU678720 *cosegregated with schizophrenia in the two families. However, we did not find a statistically significant association between the homozygous deletion of BU678720 and schizophrenia in a larger case-control sample. We did find that the prevalence of homozygous deletion of this gene was higher in the multiplex patients, but at borderline significance. We cannot totally exclude the possibility of homozygous deletion of BU678720 in the pathogenesis of familial schizophrenia. The gains and losses of genomic material assessed by array CGH seemed not to run in multiplex families. The genetic etiology in multiplex family may be better explained by other factors.

By classifying patients into 2 subgroups according to the quantity of CNV-disrupted genes, we found a correlation between the clinical psychopathological manifestations and the total burden of CNVs in DNA content. The patients with more CNVs exhibited significantly more severe negative symptoms than did those with fewer CNVs. This observation may imply that nonspecific lesions in copy numbers of the somatic genome are a discriminative property among families with schizophrenia and have applicability in predicting an elevated risk for negative symptoms. It is also interesting to note that losses are more common than gains in the patterns of numerical aberrations detected. Several genomic aberration studies of neurological disorders agreed with the finding that more frequent copy number deletions might cause disease. However, the sample size for array CGH analysis is rather small in this pilot study, and the correlation between the number of CNVs and negative symptoms may be weakened by extending the number of cases. Thus, these preliminarily results should be treated with caution.

We did find four CNS-related candidate vulnerability genes in subgroup II patients with a higher number (≥ 13) of CNVs. It is worth noting that *STK11*, found to be copy-number lost in 30% of this subgroup, has been identified as a large-scale deletion in a patient with schizophrenia and Peutz-Jeghers syndrome [36]. *RXRA *showed an exceedingly high deletion rate in five of our patient cohorts (C2, D1, E2, H1, and I) by both array CGH and qPCR. Wallen-Mackenzie et al [37] have reported that *RXRA *may contribute to schizophrenia via interaction with *Nurr1*. Additionally, several relevant reports in the literature also support its involvement in schizophrenia [38-43]. The chromosomal region containing *RXRA*, 9q34, has been associated with schizophrenia [16]. This uniformity cross-validates our results and confirms the significance of *RXRA *deletion. Two other CNS-related candidate genes, *CEBPD *and LHX5 may be associated with schizophrenia, but the results of array CGH should be treated carefully in the absence of validation by other technologies.

We intended to examine the results of array CGH by using qPCR methodology, but we do not have positive results in this regard; however, the inefficiency in validating array CGH data by using qPCR methodology has also been reported previously [18]. The chromosomal distances between the array probes and primers for qPCR may account for the discrepancies between the results of array CGH and qPCR. This may also be due to the complexity of genome sequences that influence the specificity of primers of qPCR. Thus, we still can not exclude these four CNS-related genes found in this array CGH study as schizophrenia vulnerability genes.

## Conclusions

In summary, our work further demonstrated that oligonucleotide array CGH is a useful platform for investigating the genomic aberrations of psychiatric disorders. We found the sum of altered gene dosage is coincident with severity of the negative symptoms of schizophrenia. Additionally, the CNS-related genes including CEBPD, RXRA, LHX5, and STK11 revealed by this pilot study may also provide entry points for further investigation.

## List of abbreviations

(array CGH): Array-based comparative genomic hybridization; (CNVs): copy number variations; (CNPs): copy-number polymorphisms.

## Competing interests

The authors declare that they have no competing interests.

## Authors' contributions

Author HHG designed the study and managed the literature searches. Author LCH designed the study, undertook the array CGH analysis, and wrote the draft of the manuscript. Author LCM recruited the participants, undertook the statistical analysis, and wrote the draft of the manuscript. Author WCC collected the samples. Author CSM performed the qPCR experiments. All authors contributed to and have approved the final manuscript.

## Supplementary Material

Additional file 1 **Supplementary table 1**. Information of the primers for real-time PCR. Click here for file

Additional file 2 **Supplementary table 2**. Comparison of incidence of CNVs in schizophrenics and in control subjects. Click here for file

Additional file 3 **Supplementary figure 1.** The quantitative real-time PCR (QPCR) results for potential candidate genes identified by array CGH. The fold change in gene copy number for each indicated target gene relative to the endogenous reference gene (*ATP2B4*) was compared for the genomic DNA samples from affected sib pairs with at least one showing positive results by array CGH. The fold change for each target gene and *ATP2B4 *of control sample was set at 1. The normalized fold changes were interpreted as follows: No change (0.7-1.4, white bar), homozygous loss (< 0.3, black bar), over representation (> 1.4, black bar) and ambiguous (0.3-0.7, gray bar). Click here for file

## References

[B1] Kringlen E Twin studies in schizophrenia with special emphasis on concordance figures Am J Med Genet 2000 97 4 11 10.1002/(SICI)1096-8628(200021)97:1<4::AID-AJMG2>3.0.CO;2-J 10813799

[B2] Liu H Heath SC Sobin C Roos JL Galke BL Blundell ML Lenane M Robertson B Wijsman EM Rapoport JL Gogos JA Karayiorgou M Genetic variation at the 22q11 PRODH2/DGCR6 locus presents an unusual pattern and increases susceptibility to schizophrenia Proc Natl Acad Sci USA 2002 99 3717 3722 10.1073/pnas.042700699 11891283 PMC122590

[B3] Millar JK Wilson-Annan JC Anderson S Christie S Taylor MS Semple CA Devon RS Clair DM Muir WJ Blackwood DH Porteous DJ Disruption of two novel genes by a translocation co-segregating with schizophrenia Hum Mol Genet 2000 9 1415 1423 10.1093/hmg/9.9.1415 10814723

[B4] Millar JK Christie S Anderson S Lawson D Hsiao-Wei Loh D Devon RS Arveiler B Muir WJ Blackwood DH Porteous DJ Genomic structure and localisation within a linkage hotspot of Disrupted In Schizophrenia 1, a gene disrupted by a translocation segregating with schizophrenia Mol Psychiatry 2001 6 173 178 10.1038/sj.mp.4000784 11317219

[B5] Kato C Petronis A Okazaki Y Tochigi M Umekage T Sasaki T Molecular genetic studies of schizophrenia: challenges and insights Neurosci Res 2002 43 295 304 10.1016/S0168-0102(02)00064-0 12135773

[B6] Kallioniemi A Kallioniemi OP Sudar D Rutovitz D Gray JW Waldman F Pinkel D Comparative genomic hybridization for molecular cytogenetic analysis of solid tumors Science 1992 258 818 821 10.1126/science.1359641 1359641

[B7] Macville M Schrock E Padilla-Nash H Keck C Ghadimi BM Zimonjic D Popescu N Ried T Comprehensive and definitive molecular cytogenetic characterization of HeLa cells by spectral karyotyping Cancer Res 1999 59 141 150 9892199

[B8] Iafrate AJ Feuk L Rivera MN Listewnik ML Donahoe PK Qi Y Scherer SW Lee C Detection of large-scale variation in the human genome Nat Genet 2004 36 949 951 10.1038/ng1416 15286789

[B9] Redon R Ishikawa S Fitch KR Feuk L Perry GH Andrews TD Fiegler H Shapero MH Carson AR Chen W Cho EK Dallaire S Freeman JL Gonzalez JR Gratacos M Huang J Kalaitzopoulos D Komura D MacDonald JR Marshall CR Mei R Montgomery L Nishimura K Okamura K Shen F Somerville MJ Tchinda J Valsesia A Woodwark C Yang F Zhang J Zerjal T Armengol L Conrad DF Estivill X Tyler-Smith C Carter NP Aburatani H Lee C Jones KW Scherer SW Hurles ME Global variation in copy number in the human genome Nature 2006 444 444 454 10.1038/nature05329 17122850 PMC2669898

[B10] Sebat J Lakshmi B Troge J Alexander J Young J Lundin P Maner S Massa H Walker M Chi M Navin N Lucito R Healy J Hicks J Ye K Reiner A Gilliam TC Trask B Patterson N Zetterberg A Wigler M Large-scale copy number polymorphism in the human genome Science 2004 305 525 528 10.1126/science.1098918 15273396

[B11] Zafarana G Grygalewicz B Gillis AJ Vissers LE Vliet W van de van Gurp RJ Stoop H Debiec-Rychter M Oosterhuis JW van Kessel AG Schoenmakers EF Looijenga LH Veltman JA 12p-amplicon structure analysis in testicular germ cell tumors of adolescents and adults by array CGH Oncogene 2003 22 7695 7701 10.1038/sj.onc.1207011 14576833

[B12] Lugtenberg D de Brouwer AP Kleefstra T Oudakker AR Frints SG Schrander-Stumpel CT Fryns JP Jensen LR Chelly J Moraine C Turner G Veltman JA Hamel BC de Vries BB van Bokhoven H Yntema HG Chromosomal copy number changes in patients with non-syndromic × linked mental retardation detected by array CGH J Med Genet 2006 43 362 370 10.1136/jmg.2005.036178 16169931 PMC2563232

[B13] Van Esch H Hollanders K Badisco L Melotte C Van Hummelen P Vermeesch JR Devriendt K Fryns JP Marynen P Froyen G Deletion of VCX-A due to NAHR plays a major role in the occurrence of mental retardation in patients with X-linked ichthyosis Hum Mol Genet 2005 14 1795 1803 10.1093/hmg/ddi186 15888481

[B14] Tyson C Harvard C Locker R Friedman JM Langlois S Lewis ME Van Allen M Somerville M Arbour L Clarke L McGilivray B Yong SL Siegel-Bartel J Rajcan-Separovic E Submicroscopic deletions and duplications in individuals with intellectual disability detected by array-CGH Am J Med Genet A 2005 139 173 185 16283669 10.1002/ajmg.a.31015

[B15] Sebat J Lakshmi B Malhotra D Troge J Lese-Martin C Walsh T Yamrom B Yoon S Krasnitz A Kendall J Leotta A Pai D Zhang R Lee YH Hicks J Spence SJ Lee AT Puura K Lehtimaki T Ledbetter D Gregersen PK Bregman J Sutcliffe JS Jobanputra V Chung W Warburton D King MC Skuse D Geschwind DH Gilliam TC Ye K Wigler M Strong association of de novo copy number mutations with autism Science 2007 316 445 449 10.1126/science.1138659 17363630 PMC2993504

[B16] Moon HJ Yim SV Lee WK Jeon YW Kim YH Ko YJ Lee KS Lee KH Han SI Rha HK Identification of DNA copy-number aberrations by array-comparative genomic hybridization in patients with schizophrenia Biochem Biophys Res Commun 2006 344 531 539 10.1016/j.bbrc.2006.03.156 16630559

[B17] Stefansson H Rujescu D Cichon S Pietilainen OP Ingason A Steinberg S Fossdal R Sigurdsson E Sigmundsson T Buizer-Voskamp JE Hansen T Jakobsen KD Muglia P Francks C Matthews PM Gylfason A Halldorsson BV Gudbjartsson D Thorgeirsson TE Sigurdsson A Jonasdottir A Bjornsson A Mattiasdottir S Blondal T Haraldsson M Magnusdottir BB Giegling I Moller HJ Hartmann A Shianna KV Ge D Need AC Crombie C Fraser G Walker N Lonnqvist J Suvisaari J Tuulio-Henriksson A Paunio T Toulopoulou T Bramon E Di Forti M Murray R Ruggeri M Vassos E Tosato S Walshe M Li T Vasilescu C Muhleisen TW Wang AG Ullum H Djurovic S Melle I Olesen J Kiemeney LA Franke B Sabatti C Freimer NB Gulcher JR Thorsteinsdottir U Kong A Andreassen OA Ophoff RA Georgi A Rietschel M Werge T Petursson H Goldstein DB Nothen MM Peltonen L Collier DA St Clair D Stefansson K Large recurrent microdeletions associated with schizophrenia Nature 2008 455 232 236 10.1038/nature07229 18668039 PMC2687075

[B18] Sutrala SR Goossens D Williams NM Heyrman L Adolfsson R Norton N Buckland PR Del-Favero J Gene copy number variation in schizophrenia Schizophr Res 2007 96 93 99 10.1016/j.schres.2007.07.029 17826036

[B19] Stone JL O'Donovan MC Gurling H Kirov GK Blackwood DH Corvin A Craddock NJ Gill M Hultman CM Lichtenstein P McQuillin A Pato CN Ruderfer DM Owen MJ St Clair D Sullivan PF Sklar P Purcell SM Stone JL Ruderfer DM Korn J Kirov GK Macgregor S McQuillin A Morris DW O'Dushlaine CT Daly MJ Visscher PM Holmans PA O'Donovan MC Sullivan PF Sklar P Purcell SM Gurling H Corvin A Blackwood DH Craddock NJ Gill M Hultman CM Kirov GK Lichtenstein P McQuillin A O'Donovan MC Owen MJ Pato CN Purcell SM Scolnick EM St Clair D Stone JL Sullivan PF Sklar P O'Donovan MC Kirov GK Craddock NJ Holmans PA Williams NM Georgieva L Nikolov I Norton N Williams H Toncheva D Milanova V Owen MJ Hultman CM Lichtenstein P Thelander EF Sullivan P Morris DW O'Dushlaine CT Kenny E Waddington JL Gill M Corvin A McQuillin A Choudhury K Datta S Pimm J Thirumalai S Puri V Krasucki R Lawrence J Quested D Bass N Curtis D Gurling H Crombie C Fraser G Kwan SL Walker N St Clair D Blackwood DH Muir WJ McGhee KA Pickard B Malloy P Maclean AW Van Beck M Visscher PM Macgregor S Pato MT Medeiros H Middleton F Carvalho C Morley C Fanous A Conti D Knowles JA Ferreira CP Macedo A Azevedo MH Pato CN Stone JL Ruderfer DM Korn J McCarroll SA Daly M Purcell SM Sklar P Purcell SM Stone JL Chambert K Ruderfer DM Korn J McCarroll SA Gates C Daly MJ Scolnick EM Sklar P Rare chromosomal deletions and duplications increase risk of schizophrenia Nature 2008 455 178 179 10.1038/nature07239 18668038 PMC3912847

[B20] Walsh T McClellan JM McCarthy SE Addington AM Pierce SB Cooper GM Nord AS Kusenda M Malhotra D Bhandari A Stray SM Rippey CF Roccanova P Makarov V Lakshmi B Findling RL Sikich L Stromberg T Merriman B Gogtay N Butler P Eckstrand K Noory L Gochman P Long R Chen Z Davis S Baker C Eichler EE Meltzer PS Nelson SF Singleton AB Lee MK Rapoport JL King MC Sebat J Rare structural variants disrupt multiple genes in neurodevelopmental pathways in schizophrenia Science 2008 320 539 543 10.1126/science.1155174 18369103

[B21] Wilson GM Flibotte S Chopra V Melnyk BL Honer WG Holt RA DNA copy-number analysis in bipolar disorder and schizophrenia reveals aberrations in genes involved in glutamate signalling Hum Mol Genet 2006 15 743 749 10.1093/hmg/ddi489 16434481

[B22] Xu B Roos JL Levy S van Rensburg EJ Gogos JA Karayiorgou M Strong association of de novo copy number mutations with sporadic schizophrenia Nat Genet 2008 40 880 885 10.1038/ng.162 18511947

[B23] Need AC Attix DK McEvoy JM Cirulli ET Linney KL Hunt P Ge D Heinzen EL Maia JM Shianna KV Weale ME Cherkas LF Clement G Spector TD Gibson G Goldstein DB A Genome-wide Study of Common SNPs and CNVs in Cognitive Performance in the CANTAB battery Hum Mol Genet 2009 19734545 10.1093/hmg/ddp413 PMC2773267

[B24] Kirov G Gumus D Chen W Norton N Georgieva L Sari M O'Donovan MC Erdogan F Owen MJ Ropers HH Ullmann R Comparative genome hybridization suggests a role for NRXN1 and APBA2 in schizophrenia Hum Mol Genet 2008 17 458 465 10.1093/hmg/ddm323 17989066

[B25] American Psychiatric Association Diagnostic and Statistical Manual of Mental Disorders 1994 Washington, DC: American Psychiatric Press

[B26] Andreasen NC Negative symptoms in schizophrenia. Definition and reliability Arch Gen Psychiatry 1982 39 784 788 7165477 10.1001/archpsyc.1982.04290070020005

[B27] Andreasen NC The scale for the assessment of positive symptoms (SAPS) 1984 Iowa: University of Iowa Press

[B28] Liu SK Chiu CH Chang CJ Hwang TJ Hwu HG Chen WJ Deficits in sustained attention in schizophrenia and affective disorders: stable versus state-dependent markers Am J Psychiatry 2002 159 975 982 10.1176/appi.ajp.159.6.975 12042186

[B29] Lin CC Chen WJ Yang HJ Hsiao CK Tien AY Performance on the Wisconsin Card Sorting Test among adolescents in Taiwan: norms, factorial structure, and relation to schizotypy J Clin Exp Neuropsychol 2000 22 69 79 10.1076/1380-3395(200002)22:1;1-8;FT069 10649546

[B30] Lee CH Fang CY Sheu JJ Chang Y Takada K Chen JY Amplicons on chromosome 3 contain oncogenes induced by recurrent exposure to 12-O-tetradecanoylphorbol-13-acetate and sodium n-butyrate and Epstein-Barr virus reactivation in a nasopharyngeal carcinoma cell line Cancer Genet Cytogenet 2008 185 1 10 10.1016/j.cancergencyto.2008.03.014 18656687

[B31] Lee CH Wu CC Wu YN Chiang HS Gene copy number variations in Asian patients with congenital bilateral absence of the vas deferens Hum Reprod 2009 24 748 755 10.1093/humrep/den413 19095672

[B32] Database of Genomic Variants http://projects.tcag.ca/variation/

[B33] Gene Expression Omnibus http://www.ncbi.nlm.nih.aov/geo/

[B34] Bodin L Beaune PH Loriot MA Determination of cytochrome P450 2D6 (CYP2D6) gene copy number by real-time quantitative PCR J Biomed Biotechnol 2005 2005 248 253 10.1155/JBB.2005.248 16192683 PMC1224698

[B35] PubMed http://www.ncbi.nlm.nih.gov

[B36] Kam M Massare J Gallinger S Kinzie J Weaver D Dingell JD Esufali S Bapat B Tobi M Peutz-Jeghers syndrome diagnosed in a schizophrenic patient with a large deletion in the STK11 gene Dig Dis Sci 2006 51 1567 1570 10.1007/s10620-006-9102-8 16927138

[B37] Wallen-Mackenzie A Mata de Urquiza A Petersson S Rodriguez FJ Friling S Wagner J Ordentlich P Lengqvist J Heyman RA Arenas E Perlmann T Nurr1-RXR heterodimers mediate RXR ligand-induced signaling in neuronal cells Genes Dev 2003 17 3036 3047 10.1101/gad.276003 14681209 PMC305256

[B38] Wang S Sun CE Walczak CA Ziegle JS Kipps BR Goldin LR Diehl SR Evidence for a susceptibility locus for schizophrenia on chromosome 6pter-p22 Nat Genet 1995 10 41 46 10.1038/ng0595-41 7647789

[B39] Goodman AB Congenital anomalies in relatives of schizophrenic probands may indicate a retinoid pathology Schizophr Res 1996 19 163 170 10.1016/0920-9964(96)88523-9 8789914

[B40] Goodman AB Three independent lines of evidence suggest retinoids as causal to schizophrenia Proc Natl Acad Sci USA 1998 95 7240 7244 10.1073/pnas.95.13.7240 9636132 PMC33865

[B41] Krezel W Ghyselinck N Samad TA Dupe V Kastner P Borrelli E Chambon P Impaired locomotion and dopamine signaling in retinoid receptor mutant mice Science 1998 279 863 867 10.1126/science.279.5352.863 9452386

[B42] Mena MA Casarejos MJ Bonin A Ramos JA Garcia Yebenes J Effects of dibutyryl cyclic AMP and retinoic acid on the differentiation of dopamine neurons: prevention of cell death by dibutyryl cyclic AMP J Neurochem 1995 65 2612 2620 7595558 10.1046/j.1471-4159.1995.65062612.x

[B43] Samad TA Krezel W Chambon P Borrelli E Regulation of dopaminergic pathways by retinoids: activation of the D2 receptor promoter by members of the retinoic acid receptor-retinoid × receptor family Proc Natl Acad Sci USA 1997 94 14349 14354 10.1073/pnas.94.26.14349 9405615 PMC24972

