# Supplementary material for: Genetic copy number variants in sib pairs both affected with schizophrenia
Source: J Biomed Sci. 2010 Jan 11;17(1):2. doi: 10.1186/1423-0127-17-2 (PMC2843606; doi:10.1186/1423-0127-17-2)
Supplement: Additional file 1 — Supplementary table 1. Information of the primers for real-time PCR. [file 1423-0127-17-2-S1.PDF]

Supplementary table 1. Information of the primers for real-time PCR

Gene symbol	sense primer	antisense primer
CEBPD	5'-cgtgtctctgctgaatggtggtg-3'	5'-ctccttagtctgtgatctttgctctcc-3'
RXRA	5'-tggacgccttctccatagtc-3'	5'-cagctcccctcaccactg-3'
LHX5	5'-aggtatctcgggtggctgctg-3'	5'-cggcgaccagacaaagatgagg-3'
STK11	5'-ccctttcctgtcacttccac-3'	5'-cggcgaccagacaaagatgagg-3'
BU678720	5'-tgcatctcaagttg-3'	5'-aggagaaagaa-3'
ATP2B4	5'-tgccacgaacaccactcctg-3'	5'- accctagtcccaaacttagaagcc-3'
