# Supplementary material for: Genetic copy number variants in sib pairs both affected with schizophrenia
Source: J Biomed Sci. 2010 Jan 11;17(1):2. doi: 10.1186/1423-0127-17-2 (PMC2843606; doi:10.1186/1423-0127-17-2)
Supplement: Additional file 2 — Supplementary table 2. Comparison of incidence of CNVs in schizophrenics and in control subjects. [file 1423-0127-17-2-S2.PDF]

Supplementary table 2. Comparison of incidence of CNVs in schizophrenics and in control subjects

Gene Symbol	Incidence in schizophrenia[a]	Familial incidence[b]	Incidence in normal individuals[c]	Relevant literature
PRKAG2	4/18 (22.2%)	0	1/36 (2.8%)	Mills et al. (2006)
CEBPD	4/18 (22.2%)	0	ND	
KLF4	4/18 (22.2%)	0	ND	
RXRA	5/18 (27.8%)	0	17/95 (17.5%)	Wong et al. (2007)
UBADC1	3/18 (16.7%)	0	7/95 (7.4%)	Wong et al. (2007)
LCN6	5/18 (27.8%)	0	9/270 (3.3%); 1/272 (0.4%); 3/1190 (0.3%); 1/112(0.9%)	Redon et al. (2006); Simon-Sanchez et al. (2007); Zogopoulos et al. (2007); Wong et al. (2007)
LCN8	5/18 (27.8%)	0	9/270 (3.3%); 1/272 (0.4%); 3/1190 (0.3%);2/112(1.8%)	Redon et al. (2006); Simon-Sanchez et al. (2007); Zogopoulos et al. (2007); Wong et al. (2007)
C9orf37	3/18 (16.7%)	0	3/1190 (0.3%)	Zogopoulos et al. (2007)
COL13A1	3/18 (16.7%)	0	17/50 (0.34%)	de Smith et al. (2007)
LHX5	3/18 (16.7%)	0	ND	
RHCG	3/18 (16.7%)	0	ND	
SEPT9	3/18 (16.7%)	0	1/36 (2.8%)	Mills et al. (2006)
STK11	3/18 (16.7%)	0	4/270 (1.5%); 2/1191(0.2%)	Redon et al. (2006); Zogopoulos et al. (2007)
PVR	3/18 (16.7%)	1	2/55 (3.6%)	Iafrate et al. (2004)
BU678720	4/18 (22.2%)	2	ND	
C21orf57	3/18 (16.7%)	0	3/1190 (0.3%)	Zogopoulos et al. (2007)

We confirm that all of these selected CNVs have an aberration incidence significantly higher than those found in control subjects, supporting their involvement in pathogenesis.

[a]Incidence of CNV detected in this study.

[b]Incidence of CNV observed in both siblings from a given family.

[c]Incidence in controls was obtained by searching the published CNVs listed in the Database of Genomic Variants (http://projects.tcag.ca/variation/). The content of the database represents genomic structural variation identified in healthy control samples.
