# Supplementary figures and images for: Genetic copy number variants in sib pairs both affected with schizophrenia
Source: J Biomed Sci. 2010 Jan 11;17(1):2. doi: 10.1186/1423-0127-17-2 (PMC2843606; doi:10.1186/1423-0127-17-2)

**
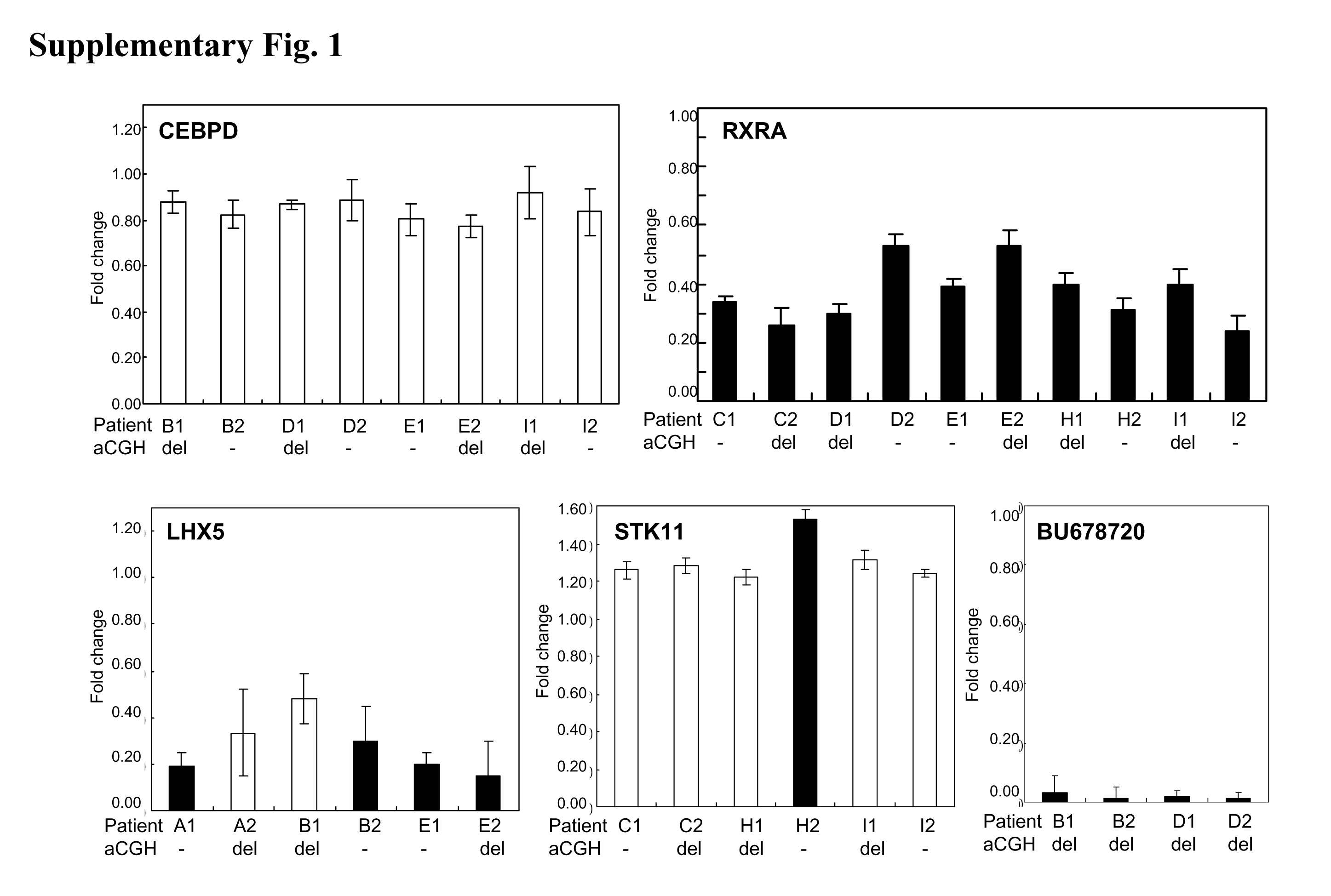
**

Supplement: Additional file 3 — Supplementary figure 1. The quantitative real-time PCR (QPCR) results for potential candidate genes identified by array CGH. The fold change in gene copy number for each indicated target gene relative to the endogenous reference gene (ATP2B4) was compared for the genomic DNA samples from affected sib pairs with at least one showing positive results by array CGH. The fold change for each target gene and ATP2B4 of control sample was set at 1. The normalized fold changes were interpreted as follows: No change (0.7-1.4, white bar), homozygous loss (< 0.3, black bar), over representation (> 1.4, black bar) and ambiguous (0.3-0.7, gray bar). [file 1423-0127-17-2-S3.DOC]
